# Diseases of Colliery Operatives in South Lancashire

**Published:** 1856-04

**Authors:** W. J. Cox


### ON THE DISEASES OF THE COLLIERY OPERATIVES IN A PART OF SOUTH LANCASHIRE, AND THEIR PREVENTIBLE CAUSES.

By WILLIAM I. COX, M.R.C.S.

THE mission of the modern, the true physician, is as much to prevent as to cure disease. Prophylaxis is as great a testimony of skill and scientific research as therapeia; whilst it affords a surer evidence of that disinterested and Christian philanthropy, which should characterise the professors of the noble calling of physic.

No medical practitioner resident for any length of time in the midst of a mining or manufacturing district, can fail to remark and to deplore (provided he be possessed of the requisite powers of observation and of the ordinary feelings of humanity) the vast amount of evils in the life of the industrial classes, and the large proportion of disease springing from causes associated with their daily life, toils, and habits, which, if not altogether removable, might at least be lessened and mitigated by judicious measures.

Holding the appointment of medical officer to an extensive colliery in the vicinity of Wigan, I have had and still have ample opportunities of observing the principal maladies affecting colliery operatives, of tracing their predisposing and exciting causes, and of trying to estimate to what extent these are removable. I propose then, in a very condensed form, to consider *seriatim*—

1. The DISEASES* of colliery operatives:

---

* It is scarcely necessary to remark, that in this little essay I shall not treat of any of the *accidents* frequently occurring in coal-mines (explosions from gases, etc.), nor of their disastrous results.

2. The CAUSES of such diseases: and

3. REMEDIAL OR PREVENTIVE MEASURES.

1. DISEASES. *a. Pulmonary* diseases. Bronchitis (acute and chronic), asthma (vesicular emphysema), and tubercular phthisis are very prevalent. Chronic bronchitis, often complicated or associated with emphysematous lungs and enlargement of the right cavities of the heart, is perhaps the most frequent chest affection. Asthma (I merely wish to indicate by this much abused term the more or less permanent dyspnœa, consequent on various pathological states of the pulmonary tissue and pleural cavity) is so common, as to be designated by some writers the "scourge of the coal mine." Tubercular deposit and its effects are very frequently met with amongst colliers. The local irritation of the pulmonary tissue, excited by the almost perpetual inhalation of coal-dust, etc., contributes much towards the developement of phthisis; by awakening into fatal activity the dormant seeds of disease, and by favoring local congestion around the air-cells, as well as by inducing bronchial spasm and its secondary results. That peculiar morbid condition of lung, known as *spurious melanosis*, or *anthracosis*, but which might with more propriety of nomenclature be termed black or *carbonaceous infiltration*, is frequently discovered in the few and scattered autopsies which the ignorance and prejudices of this class of the community permit us to make. The "black spit" is the popular cognomen of the chief sign of this disease during life. This remarkable pathological phenomenon was first noticed by Pearson, was ably described by Laennec in 1806, and subsequently by Dr. William Thomson (*Med.-Chirurg. Trans.*). Whether, as suggested by Dr. Watson, the carbonaceous matter acts injuriously *only* on those who are already, by heritage or otherwise, unsound in the lungs or prone to pulmonary consumption, may be regarded as still a matter of uncertainty. I think it highly probable that such is the case. *Sed lis adhuc sub judice est.* It seems, however, an ascertained fact, that when the deposit has proceeded to any considerable extent, it acts as a foreign body; irritating the delicate tissue, and, of course, hastening the developement of disease.

*β. Pleuritis*, acute and chronic, with its ordinary results, hydrothorax and empyema, is of frequent occurrence. The men and boys employed at the "brow", as it is termed, *i.e.*, in the open air at or near the pit's mouth, are sufferers in greater proportion from these affections than the pitmen themselves. It is not difficult to explain this. They are equally liable to the inhalation of coal-dust and of mephitic vapours from the pit-fires, and are, moreover, exposed to wet and cold and all vicissitudes of weather.

γ. *Heart* disease, in its various forms and pathological phases, is very common, and is, I believe, on the increase amongst the miners. Pericarditis and endocarditis are frequently seen, associated with attacks of acute rheumatism; valvular lesions naturally follow, as the distressing results of repeated attacks of these maladies. Hypertrophy (especially of the right ventricle) and thickening of the free borders of the semilunar valves, both aortic and pulmonary (probably from what have been termed vegetations or granulations), are among the most frequent pathological conditions.

δ. *Fibrous* and *synovial rheumatisms* are by no means unfrequent; the former leading to the structural cardiac lesions already referred to, and ultimately to death by asthenia or dyspnœa; the latter to stiff and contracted joints. The former is of most frequent occurrence. Muscular rheumatism also in its various forms, particularly lumbago, is very common.

ε. *Renal* disease, chiefly morbus Brightii or albuminuria, is not rare. Sometimes it would seem to date from the mechanical injuries to the back and loins, to which miners are necessarily very subject: and still more frequently it appears to be in some measure induced by long persistence in the unnatural and irksome position which these men are compelled, under certain circumstances, to assume during labour; crouching down, doubled up, as it were, in a low-roofed chamber, yet obliged to exert considerable muscular effort in this posture. It is not surprising from this, that the kidneys should ultimately suffer. Most commonly, however, the renal mischief is associated with hypertrophied heart, and doubtless might be traced to a common cause—habitual intemperance. Of course, all these exciting causes may be conjointly in operation.

ζ. *Cutaneous* diseases, such as have for their especial exciting cause want of cleanliness, are often present—*prurigo*, *scabies*, and *impetigo*, are the most frequent visitants.

On the whole, pulmonary and cardiac diseases have most victims.

2. Causes. The remediable or mitigable causes of the diseases above enumerated are as follow, and may be imperfectly classed as,—A, primary or exciting causes; and B, secondary or predisponent causes.

A. *a. Perpetual neglect of personal cleanliness*, as regards removal of cutaneous filth, etc., so that the due function of the skin is in constant abeyance. This neglect is carried to an almost incredible extent.

*b. Habitual intemperance* in the use of alcoholic liquors. Drunkenness, according to recent statistics, is more rife in the colliery districts of South Lancashire, than in any other part of England. In the immediate district wherein these observations are chiefly made, the prevalence and extent of this destructive vice is truly appalling. The township of Hindley, in the borough of Wigan, containing, according to the last census, a population of 7,800, has fifty-two houses for the sale of beer, whereof eleven are also licensed for spirits.

*c. Entire neglect of the most obvious precautions against the effects of the great vicissitudes of temperature* to which the pitmen are exposed, in going to and from the scene of their labours; the workings of the mine having, of course, a nearly uniform temperature, independent of the weather or seasons.

B. *a. Want of proper ventilation* in the dwellings of the operatives, and the consequent respiration of impure and vitiated air.

*b. Deficient water supply.* In this district, which, as stated above, contains between seven thousand and eight thousand souls, and also in the adjoining township of Abram, there may be said to be no water-supply at all. The town of Hindley itself is dependent for all water-supply on some five or six semi-public pumps, and about as many open wells (dry in summer). I had nearly forgotten, however, to mention the resources of a filthy and polluted stream, running through the centre of the town, of which many of the poorer class of inhabitants avail themselves. The supply in the more rural district of Abram is equally inadequate. Foul stagnant pools, and wells few and far between, are the only sources. Need I say that dirt, and its attendant profligacy, reign supreme in this neglected locality?

*c. Absence of education.* A very small portion only of those employed in the coal-mines can read or write. From statistics which I have myself collected, and also from other trustworthy sources, I find that among the colliers in this district, one only in four can read, and that but one in six is able both to read and write.

*d. The gross immorality* which prevails among the colliery operatives. Unbounded licentiousness in the intercourse between the sexes, which scorns every restraint of morals or decency, is so common, that it is rare indeed to find a wife who has not been a mother previous to her marriage, and that, too, whilst yet in her teens. Concubinage and unrestrained conversation are the order of the day. The indecent and demoralising practice of the employment of women at the "brow", dressed in male attire, no doubt contributes much towards this deplorable social state. But, be the cause what it may, no observer of human nature can question or underrate the vast influence which such a condition must exert over the *habits* of the individual, or its reaction upon his or her physical welfare. Self-respect being lost, personal negligence and indifference to bodily decorum and to the healthful condition of dwellings soon follow; aversion to cleanliness, and love of wallowing in filth and in physical and moral pollution, is soon engendered. The habitual infringement of the *moral* law becomes, in short, directly and indirectly a prolific source of *bodily* disease and ruin.

*e. Entire absence of intellectual converse* and associations for mental or moral progress. But little idea of enjoyment is entertained, save that suggested by a degraded sensualism. No public social meetings, no scientific association, no mechanics' institute (at least in this locality), offer any antidote to the beershop, or any attraction in lieu of the glass of ale.

*f.* Lastly, I may be allowed to mention, a sad want of harmony with other classes of men. It is notoriously a fact, that colliers live almost apart from society. They are a class *per se*, and have little sympathy with their fellowmen employed in other pursuits.

Hindley in Wigan, Lancashire, Feb. 1856.

(*To be continued.*)

